# Meta-analyses of 4 *CFTR* variants associated with the risk of the congenital bilateral absence of the vas deferens

**DOI:** 10.1186/2043-9113-4-11

**Published:** 2014-08-21

**Authors:** Xuting Xu, Jufen Zheng, Qi Liao, Huiqing Zhu, Hongyan Xie, Huijuan Shi, Shiwei Duan

**Affiliations:** 1 Zhejiang Provincial Key Laboratory of Pathophysiology, School of Medicine, Ningbo University, Ningbo, Zhejiang 315211, China; 2 China National Population and Family Planning Key Laboratory of Contraceptive Drugs and Devices, Shanghai Institute of Planned Parenthood Research (SIPPR), Shanghai 200032, China; 3 China National Population and Family Planning Key Laboratory of Contraceptive Drugs and Devices, SIPPR, Fudan University, Shanghai 200032, China

**Keywords:** Congenital bilateral absence of the vas deferens, Meta-analysis, *CFTR* gene, 5T, ΔF508, M470V

## Abstract

**Aims:**

The aim of our study was to evaluate the relationship between four *CFTR* variations and the congenital bilateral absence of the vas deferens (CBAVD).

**Methods:**

A systematic search was performed in the literature databases for the case–control studies of *CFTR* variations with the risk of CBAVD. A total of 29 studies among 1139 controls and 1562 CBAVD patients were gathered for the meta-analyses of three commonly tecsted variations (5T, ΔF508 and M470V) with CBAVD.

**Results:**

Our meta-analyses observed significant associations between CBAVD and all the three variations, including 5T (P < 0.001, OR = 8.35, 95% CI = 6.68-10.43), M470V (P = 0.027, OR = 0.74, 95% CI = 0.60-0.91) and ΔF508 (P < 0.001, OR = 22.20, 95% CI = 7.49-65.79).

**Conclusion:**

In the current study, we demonstrated a significant association between *CFTR* variations and CBAVD. Our results showed that the 5T variation was a risk factor of CBAVD in French, Spanish, Japanese, Chinese, Iranian, Indian, Mexican and Egyptian populations. *CFTR* ΔF508 was another important risk factor in Caucasians, including Slovenians, Canadians, Iranians, and Egyptians. In addition, M470V was a protective factor among French, Chinese, Italian and Iranian populations.

## Introduction

Congenital bilateral absence of the vas deferens (CBAVD, MIM: #277180) is one of the abnormal forms in the male reproductive system, causing obstruction to sperm outflow into the urethra. CBAVD is responsible for 1-2% of male infertility [1]. CBAVD was widely considered as an atypical symptom of cystic fibrosis (CF, MIM: #219700) [2,3], which was a severe recessive disease characterized by obstructive chronic lung disease, pancreatic disease and abnormal concentrations of electrolytes in the sweat in clinical studies [4]. Approximately 97% of the male CF patients also suffered from CBAVD [5].

CF is a severe genetic disease with a incidence of 1/2500 births every year in Caucasians [6]. CF patients are often carriers of CF*TR* gene variations [7] that were originally found in CBAVD patients [8,9]. The *CFTR* gene encodes a glycosylated transmembrane protein that is a chloride channel conducts the regulation of other transport pathways. *CFTR* was widely expressed in epithelial cells of exocrine tissues, such as the sweat glands, lungs, and vas deferens [10]. Published studies have shown evidence that abnormal expression of *CFTR* contributed to the dysfunction of body organs, including the sweat glands, respiratory system and reproductive systems. CBAVD is one of the generally acknowledged monosymptomatic CFTR-related disorders [11].

Located on chromosome 7q31.2, the *CFTR* gene contains 27 exons and 1945 polymorphisms according to the Cystic Fibrosis Variations Database [12]. There are four commonly tested variations (ΔF508, 5T, M470V and R1117H) in CBAVD.

The abnormal fold of the △F508-CFTR protein triggered the activity of proteasomes for degradation, which caused a lower open probability than normal [13]. Another common variations, IVS8-Tn, had three forms containing five, 7 seven, or 9 nine thymidine residues in this locus, which is considered an incomplete penetrance [14]. The 5T allele especially produces high levels of *CFTR* transcript without exon 9, resulting in variable phenotypes which were observed in CBAVD or mild CF patients [4]. Experimental evidence indicates that 5T carriers have a decreased splicing efficiency of intron 8 [15,16]. M470V (1540A/G in exon 10) is in a strong linkage disequilibrium with the 5T locus in the CBAVD population, and has become a commonly tested locus [17-19]. The 470V-CFTR protein has lower function than the 470M-CFTR protein [20]. There were arguments on the role of M470V in the CBAVD, although M470V might increase the penetrance of 5T and lead to an obvious association between 5T and Val470 alleles in CBAVD but not in fertile males.

CBAVD incidence is rare (1-2%) in the patients with male infertility [1]. Most genetic studies only involved a few number of individuals. Meanwhile, there was large ethnic difference for the CFTR variations that were more frequent in Caucasians than in other populations, such as Asians. Meta-analysis has an advantage in the sample size by pooling all the relational studies. In the present study, we performed a comphrehensive search for the eligible case–control studies on the association between CBAVD and three *CFTR* variations, including F508del, IVS8-Tn, and M470V. The goal of our study was to evaluate the overall contribution of the three *CFTR* variations to the risk of CBAVD in different ethnic groups through systematic meta-analyses.

## Materials and methods

### Literature search and study selection

The search terms including CBAVD, SNP or variations and *CFTR* gene were used in the literature search on the PubMed. We investigated all publications on the association between *CFTR* variants and CBAVD disease from 1993 to 2013. Reference lists in the harvested literatures were explored for additional relevant studies suitable for inclusion in our meta-analysis. The selection criteria of studies for our meta-analysis were as follows: (1) CBAVD patients with typical CF were excluded; (2) complete data with allele or genotype in the case–control studies was available; (3) only CBAVD diagnosis for studies with physical examination, semen analysis and ultrasonography were considered. The retrieved information consisted of author, year of publication, ethnicity, and the number of participants with different alleles.

### Statistical analysis

All the eligible case–control studies were involved in the meta-analysis done by Stata software (version 11.0, Stata Corporation, College Station, TX) [21]. Total odd ratios (OR) and 95% confidence intervals (CIs) were estimated to assess the association between *CFTR* variations and CBAVD risk. Cochran’s Q statistic and I^2^ test were used to test heterogeneity in our study. The Fixed-effect model was used for meta-analysis with moderate heterogeneity (I^2^ < 50%). Otherwise, a random-effect model was adopted for the meta-analysis with significant heterogeneity (I^2^ > =50%) [22,23]. Z-test with a two-sided P value < 0.05 was used to determine the significance of the total ORs. The publication bias in the meta-analysis was evaluated by the funnel plot and Egger’s test. Summary analysis of the overall frequency of all the studies was executed to show the frequency for all fou variations [24]. Subgroup analysis was performed by ethnicity to evaluate the effect of different ethnicity on total ORs.

## Results

**Figure 1:**
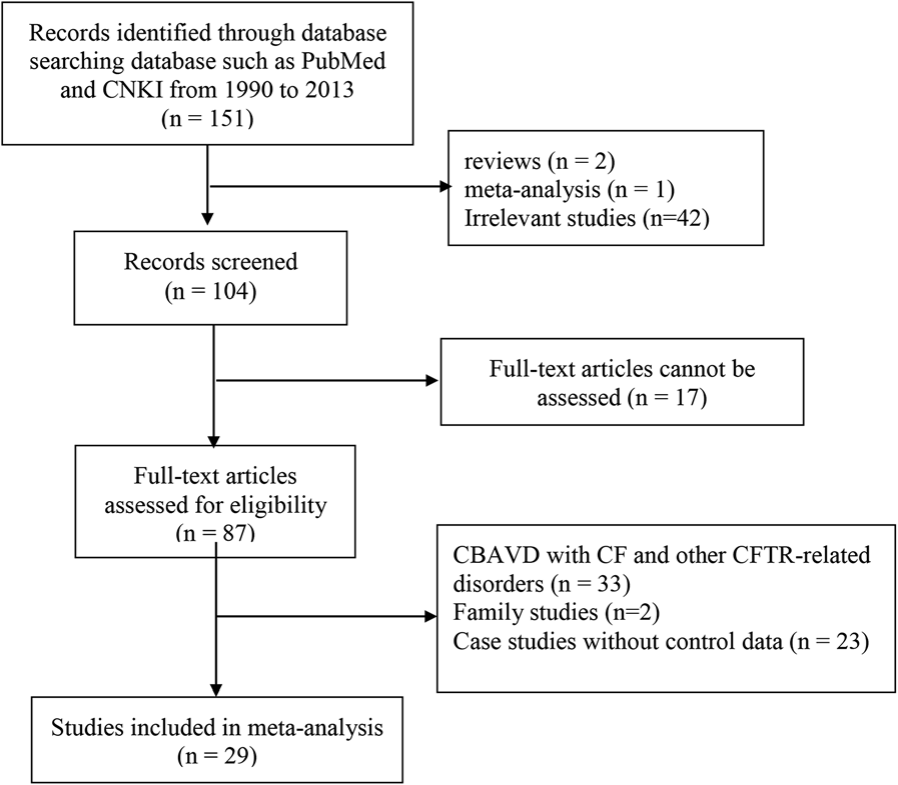
Flow diagram of the meta-analyses.

Our comprehensive literature search returned a total of 149 articles on the association between *CFTR* and CBAVD from PubMed and two studies from CNKI. A total of 104 studies was harvested after being filtered by their titles and abstracts. Among those articles, we removed four family studies and three reviews or meta-analysis. Consequently, 26 studies without obtainable allele or genotype data were removed. Among them, there were 29 studies on three CFTR variations. Our meta-analyses of three CFTR variations were performed among 1139 cases and 1563 controls (Figure 1, Table 1).

**Table 1:** The characteristics of the enrolled mutation for OR meta-analysis

**Mutation**	**Author**	**Ethnic**	**Year**	**Total (case/control)**	**Allele (case/control)**	**Total1**	**Allele1**	**Total2**	**Allele2**
T5	Costes	French	1995	45/66	23/7	90	23	131	7
	Chillon	Spanish	1995	102/46	42/5	204	42	92	5
	de Meeus	French	1998	60/133	20/11	120	20	266	11
	Casals	Spanish	2000	104/200	41/20	208	41	400	20
	Ravnik-Glavac	Slovenia	2001	7/95	3/8	14	3	190	8
	Anzai	Japanese	2003	19/53	11/0	38	11	106	0
	Grangeia	Caucasian	2004	31/114	17/8	62	17	228	8
	Wu, C. C.	Chinese	2004	27/46	34/5	54	34	92	5
	Wu, C. C.	Chinese	2005	36/23	21/1	72	21	46	1
	Radpour, R.	Iranian	2006	106/43	55/0	212	55	86	0
	Wilschanski	Canadian	2006	60/31	22/2	120	22	62	2
	Radpour, R.	Iranian	2007	112/84	61/0	224	61	168	0
	Costa	NA	2008	68/62	33/6	136	33	124	6
	Chiang	Chinese	2009	63/86	42/6	126	42	172	6
	Sharma	Indian	2009	39/50	21/5	78	21	100	5
	Sachdeva	Indian	2011	35/50	19/1	70	19	100	1
	Hussein	Egyptian	2011	30/30	16/3	60	16	60	3
	Saldana-Alvarez	Mexican	2012	16/103	1/3	32	1	206	3
	LU	Chinese	2013	109/104	97/28	218	97	208	28
ΔF508	Ravnik-Glavac	Slovenia	2001	7/95	2/3	14	2	190	3
	Kusic, J.	NA	2002	10/11	5/1	20	5	22	1
	Wilschanski, M.	Canadian	2006	60/31	30/1	120	30	62	1
	Radpour, R.	Iranian	2007	112/84	28/0	224	28	168	0
	Hussein, T. M.	Egyptian	2011	30/30	12/0	60	12	60	0
M470V	de Meeus, A.	French	1998	60/133	52/158	120	52	266	158
	Wu, C. C.	Chinese	2005	36/53	35/61	72	35	106	61
	Stuppia, L.	Italian	2005	13/54	4/38	26	4	108	38
	Radpour, R.	Iranian	2006	106/43	69/21	212	69	86	21
	Du, Qing	Chinese	2013	95/135	99/158	190	99	270	158

**Figure 2:**
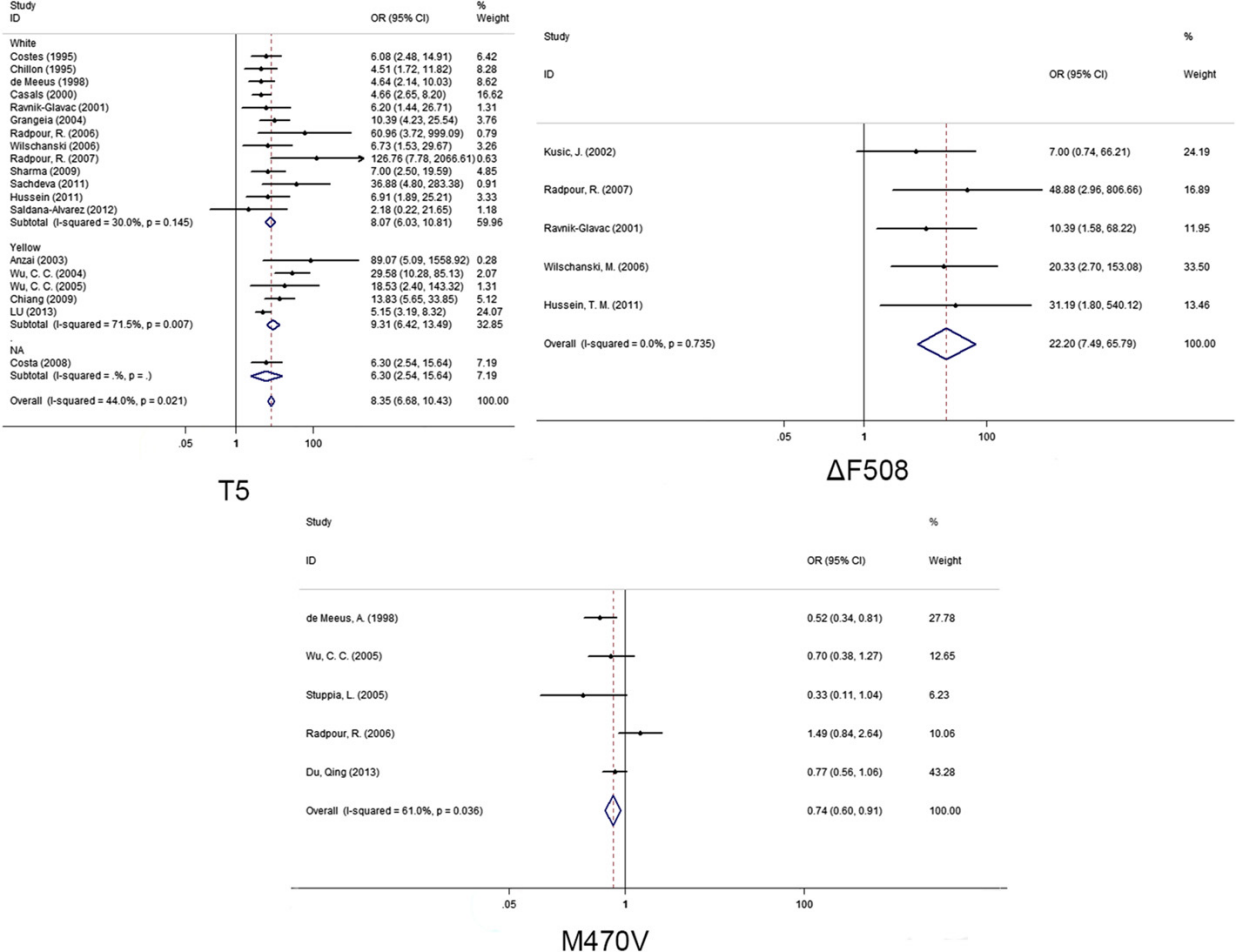
Forest plots for the relationship between 3 variations and CBAVD in the meta-analyses and subgroup meta-analyses.

A strong association between the *CFTR* 5T variant and CBAVD was observed in the meta-analysis of 19 studies among 1069 cases and 1419 controls (OR = 8.35, 95% CI = 6.03-10.81, Figure 2). Our results also found that ΔF508 was a risk factor of CBAVD (OR = 22.2, 95% CI = 7.49-65.79, Figure 2). Our meta-analysis showed the M470V variation was associated with CBAVD (OR = 0.74, 95% CI = 0.60-0.91, Figure 2). Future study is required to explore whether there are other causal variants in high linkage disequilibrium with M470V.

**Figure 3:**
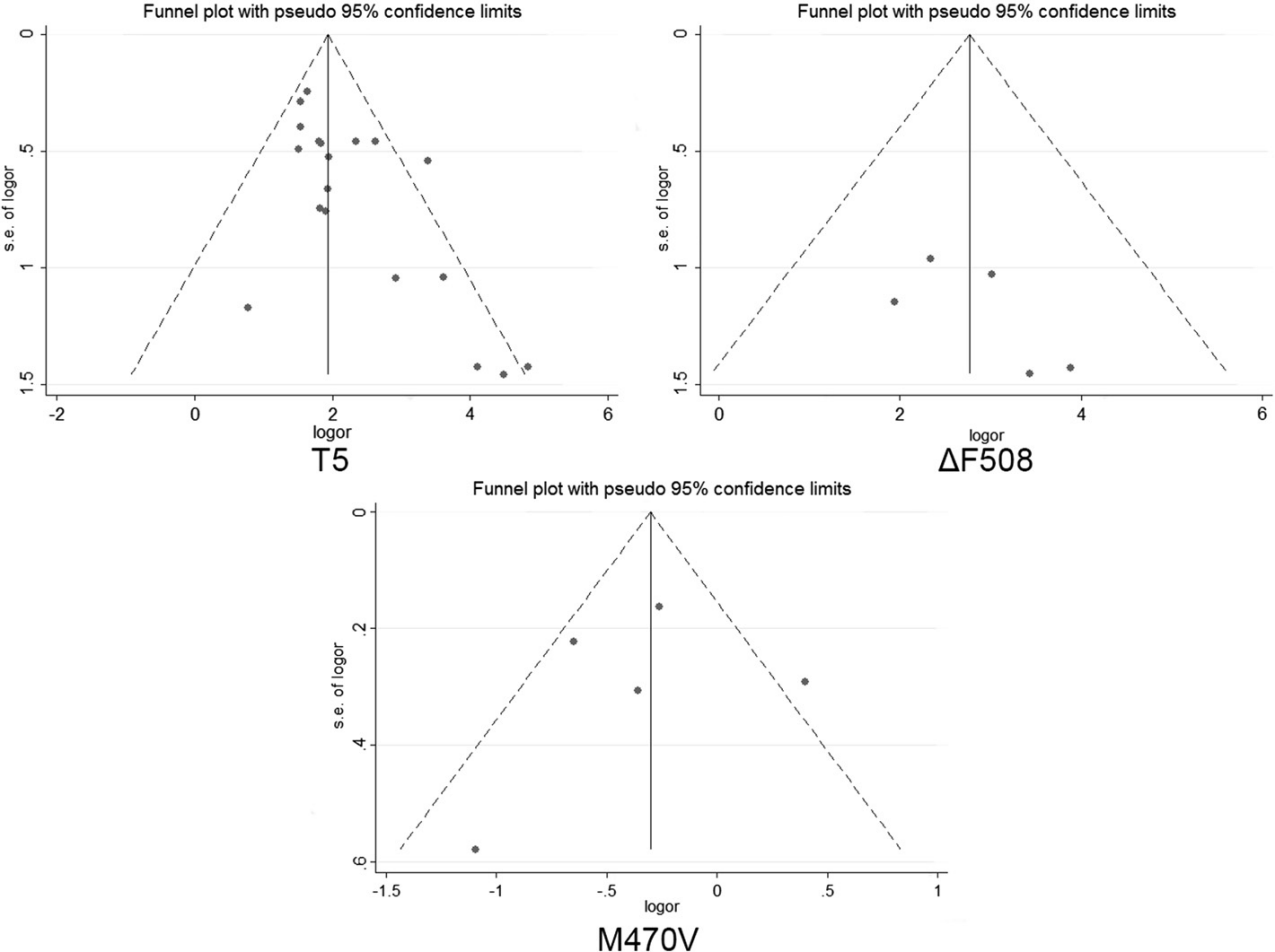
Funnel plots for the relationship between 3 variations and CBAVD in the meta-analyses.

**Figure 4:**
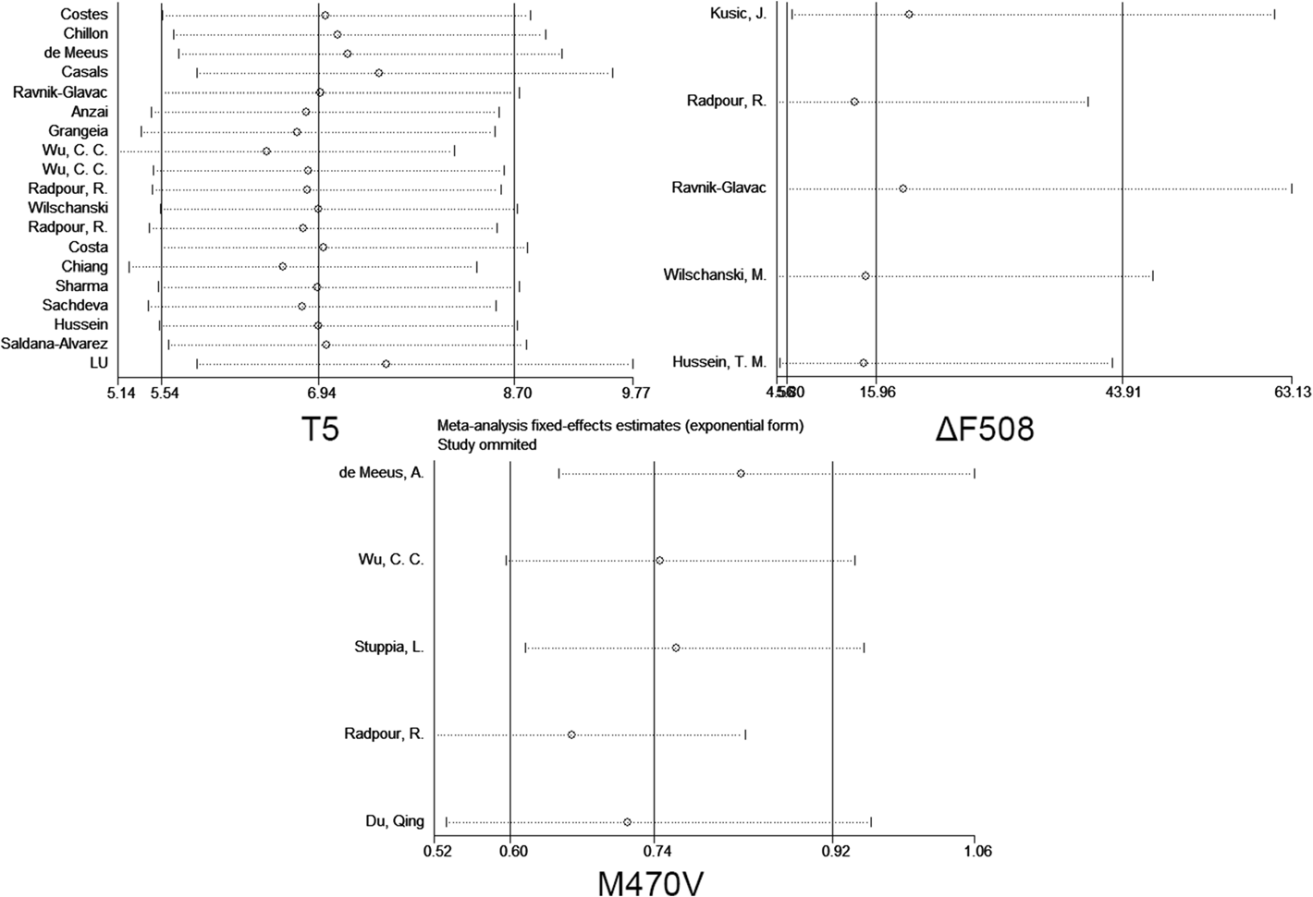
Meta influence analysis for 3 variations.

As shown in Figure 2, moderate heterogeneity was observed for *CFTR* 5T (I^2^ = 44.0%), M470V (I^2^ = 61.0%) and ΔF508 (I^2^ = 0.00%). Furthermore, subgroup meta-analyses of the 5T variations by ethnicity showed that the heterogeneity mainly came from Asians (I^2^ = 71.5%) instead of Caucasians (I^2^ = 30.0%). Our results indicated that 5T was a risk factor of CBAVD in Caucasians (OR = 8.07, 95% CI = 6.03-10.81, P = 0.00, Figure 2), as well as in Asians (OR = 9.31, 95% CI = 6.42-13.49, P = 0.00, Figure 2). As shown in the funnel plot, no obvious publication bias was observed for the meta-analyses (Figure 3). The results of meta-analyses were robust after the removal of individual studies (Figure 4).

## Discussion

In the present study, we carried out a systematic overview of the studies for a genetic link with CBAVD. Significant evidence was observed (that showed) the strong association with CBAVD onset with the risk of CBAVD for all the three variations, including 5T (OR = 8.35, 95% CI = 6.03-10.81, Figure 2), M470V (OR = 0.74, 95% CI = 0.60-0.91, Figure 2) and ΔF508 (OR = 22.2, 95% CI = 7.49-65.79, Figure 2).

Among the tested variations, M470V was the only one with the protective role of CBAVD. V470 carriers tended to have a higher fertility than other carriers, suggesting that there might be competing and evolutionary forces acting on the *CFTR* gene [25]. Furthermore, the interaction of the variations might affect the risk of CBAVD [26,27]. In addition, the haplotypes of these variations can significantly contribute to the susceptibility of CBAVD alone or together with the *SPINK1* locus [11]. Further detailed studies are required to provide evidence that independent CBAVD rather than CBAVD with CF is associated with *CFTR* variations.

Several limitations in our meta-analyses were taken into consideration as followed. Firstly, some studies did not reveal whether CBAVD patients were isolated CBAVD patients or patients with CF symptoms for the incomplete information. This might potentially interfere with the results of our meta-analyses. Secondly, the power of our meta-analyses is still low due to low allele frequency of these variations. Under a moderate risk of OR (OR = 1.2), power analysis showed a lack of power in the meta-analyses, including 5T (47.3%), ΔF508 (7.1%) of LPL, and M470V (52.1%). Thirdly, there was a lack of haplotype information among these loci in most of involved studies. Reports have shown there were interactions among these variations [28-30]. We discontinued the further analysis for examining variables, including interaction between the two or three loci.

In conclusion, the present study demonstrated an association between *CFTR* variants and CBAVD risk. Our meta-analysis among 1069 cases and 1418 controls showed that 5T was a risk factor of CBAVD in the combined populations, including French, Spanish, Japanese, Chinese, Iranian, Indian, Mexican and Egyptian. Meta-analysis among 60 cases and 133 controls confirmed that *CFTR* ΔF508 was another important risk factor in Caucasian populations including Slovenian, Canadian, Iranian and Egyptian. In addition, M470V was shown to be a protective factor by our meta-analysis among 10 cases and 11 controls from French, Chinese, Italian and Iranian populations.

## Abbreviations

SNP: Single nucleotide polymorphism; CBAVD: Congenital bilateral absence of the vas deferens; CF: Cystic fibrosis; OR: Odds ratio; 95% CI: 95% confidence interval; I^2^: Inconsistency index.

## Competing interests

All authors declare no competing financial interests.

## Authors’ contributions

XTX drafted the manuscript; JZ and QL were responsible for analysis; HZ and HX participated in literature search. SJ and SD carried out language editing. All authors read and approved the final manuscript.
